# Knowledge atlas of the involvement of glutamate and GABA in alcohol use disorder: A bibliometric and scientometric analysis

**DOI:** 10.3389/fpsyt.2022.965142

**Published:** 2022-08-12

**Authors:** Zhanzhang Wang, Xiuqing Zhu, Xiaojia Ni, Yuguan Wen, Dewei Shang

**Affiliations:** Department of Pharmacy, The Affiliated Brain Hospital of Guangzhou Medical University, Guangzhou, China

**Keywords:** glutamate, GABA, alcohol use disorder, bibliometrics, scientometrics, visualization

## Abstract

**Introduction:**

Abnormal neurotransmission of glutamate and γ-aminobutyric acid (GABA) is a key characteristic of alcohol-related disorders. To track research output, we conducted a bibliometric analysis to explore the current status and trends in this field over the past decades.

**Methods:**

Studies related to neurotransmitters and alcohol use disorder published in English from 2005–2021 were retrieved from the Web of Science Core Collection and Scopus databases. The R–*bibliometrix* package was used for a descriptive analysis of the publications. Citespace, WOSviewer, and R–*bibliometrix* were used to construct networks of countries/institutions/authors based on co-authorship, co-citation analysis of cited references and co-occurrence as well as burst detection of keywords.

**Results:**

A total of 4,250 unique articles and reviews were included in the final analysis. The annual growth rate of publications was 5.4%. The USA was the most productive country in this field, contributing nearly half of the total documents. The top ten most productive institutions were all located in the USA. The most frequent worldwide collaboration was between the USA and Italy. The most productive and influential institution was the University of California. The author contributing the most productions to this field was Marisa Roberto from the Scripps Research Institute. The top co-cited reference was a review titled “Neurocircuitry of addiction.” The top journal in terms of the number of records and citations was *Alcoholism: Clinical and Experimental Research*. Comprehensive analyses have been conducted over past decades based on co-cited reference analysis, including modulators, transporters, receptor subtypes, and animal models. In recent years, the research frontiers have been shifting to the identification of risk factors/biomarkers, drug development for alcohol use disorder, and mechanisms related to alcoholic and non-alcoholic fatty liver.

**Conclusion:**

Our bibliometric analysis shows that glutamate and GABA continue to be of interest in alcohol use disorder. The focus has evolved from mechanisms and medications related to glutamate and GABA in alcohol use disorder, to novel drug development, risk factor/biomarker identification targeting neurotransmitters, and the mechanisms of related diseases.

## Introduction

Alcohol is the oldest and most extensively used addictive substance. Acute and chronic consumption of alcohol may lead to alcohol dependence, alcohol withdrawal syndrome and other alcohol-related disorders. Meanwhile, alcohol intake is a risk factor for a variety of diseases such as hypertension, diabetes, and liver cirrhosis. The harmful use of alcohol has become a severe global threat, leading to more than 3.3 million deaths every year (1). In some countries, the all-cause mortality rate of alcohol is as high as 10% (1). Alcohol use disorder is a complex dynamic process, involving adaptive changes related to the neuroendocrine system, neurotransmitters [γ-aminobutyric acid (GABA), glutamate, monoamines], neuropeptides [corticotropin releasing factor (CRF), neuropeptide Y (NPY)] and ion channels (voltage-gated ion channels, small-conductance Ca^2+^-activated K^+^ channel, big potassium ion channel). The disruption of neurochemical homeostasis not only facilitates symptoms of alcohol-related disease, but also augments the sensitivity to harmful drinking behavior.

Glutamate is the major neurotransmitter for excitatory synaptic signaling in the brain. Numerous studies in recent years have suggested a complex influence of alcohol on glutamate receptors, glutamate transporters, and synaptic glutamate homeostasis in different brain regions and neurocircuitries, as well as corresponding behavioral changes. Glutamate receptors are categorized into metabotropic glutamate receptors (mGluRs) and ionotropic glutamate receptors (iGluRs), regulating slow and rapid glutamatergic neurotransmissions, respectively. Acute exposure and chronic exposure to alcohol have opposite effects on N-methyl-D-aspartate receptor (NMDAR)-mediated synaptic plasticity and glutamatergic synaptic transmission (2). Chronic exposure enhances the NMDAR-mediated synaptic plasticity and glutamatergic synaptic transmission, while acute alcohol exposure exerts an inhibitory effect. Alcohol also increases subunits [GluA1, GluA2, and GluA3 (3)] of α-amino-3-hydroxy-5-methyl-4-isoxazolepropionic acid receptor (AMPAR) expression and its synaptic localization. In general, acute alcohol exposure inhibits neuronal excitability, while chronic alcohol exposure increases the functioning of iGluRs. By contrast to iGluRs, alcohol exposure has a modest effect on mGluRs. Expression of group I mGluRs (mGluR1 and mGluR5), predominantly localized in the post-synaptic neurons regulating slow excitatory neurotransmission, is upregulated by alcohol. Meanwhile, expression of mGluR2/3, which plays a critical role in reducing the release of glutamate from pre-synaptic glutamatergic neurons, is downregulated. Agonism of group I mGluRs and/or antagonism of group II mGluRs by alcohol restores glutamate homeostasis in the brain. Accumulating evidence indicates that alcohol exposure alters the expression and functions of the glutamate transporters. Excitatory amino acid transporters (EAAT1-5) are region- and cell subtype-specific, and are responsible for removing glutamate from the synapse into the glial cells to maintain glutamate homeostasis. The EAAT1 (GLAST) and EAAT4 are mainly expressed in the cerebellum. The EAAT2 (GLT-1) is largely located in the forebrain. The EAAT3 is primarily expressed in neurons more homogeneously, and the EAAT5 is limited to ocular cells. The cystine–glutamate antiporter (xCT) is also important in extra-synaptic glutamate homeostasis, by exchanging extracellular cystine for intracellular glutamate in astrocytes. The expression of GLT-1 and xCT is downregulated by chronic alcohol exposure (3), leading to a significant increase in extracellular glutamate levels. Given that the major glutamate transporters GLAST, GLT-1, and xCT are predominantly expressed in astrocytes, astrocytes are inevitably important for glutamate homeostasis in the brain. Alcohol exposure dysregulates glutamatergic neurotransmission *via* glutamate receptors and transporters in the mesocorticolimbic brain regions, and modulations to these targets attenuate alcohol-seeking behavior [for a review, see (3)].

GABA is the major inhibitory neurotransmitter in the brain, underlying many alcoholic behavioral changes including anxiolytic, anticonvulsant, sedative-hypnotic, cognitive-impairing, and motor-incoordinating actions (4). Alcohol exerts direct and indirect effects on GABA receptors and GABA release. There are two classes of GABA receptors: GABA_A_ (ligand-gated ion channels) and GABA_B_ (G-protein-coupled receptors). GABA_A_Rs are postsynaptic pentameric complexes assembled with various subunits. The functions of GABA_A_R subtypes are mainly defined by the specific α subunit: α1, α2, and α4 subunits are the most relevant subunits in alcohol dependence (4). Low-dose alcohol enhances the tonic inhibition mediated *via* multiple subunits such as α4/6βδ- and α1δ-GABA_A_ (5), which may be involved in the maintenance of ethanol self-administration. Alcohol also mediates post-translational modification of GABA_A_ receptors, including phosphorylation of GABA_A_ receptor subunits by protein kinase C (PKC) (6), and alters GABA_A_ expression by protein kinase A (PKA). The cAMP-PKA signaling pathway also plays an important role in the neurobiological responses and behavioral actions induced by alcohol. Apart from direct modulation of GABA_A_Rs, alcohol may influence functions of GABA_A_Rs in an indirect way *via* neurosteroids (7). Accumulating evidence also shows that alcohol increases GABAergic synaptic transmission by increasing presynaptic GABA release from vesicles, especially in the central amygdala (CeA) (8). The utility of alcohol in facilitating GABA transmission may be limited by GABA feedback onto presynaptic GABA_B_. GABA transporters (GAT1-3) are responsible for removing GABA from the synaptic cleft. GAT-1 is predominantly found in axon terminals and glial cells, and GAT-3 is exclusively located on glial cells (9). Recently, Augier et al. (10) found that GAT-3 expression was selectively decreased in the CeA of alcohol-choosing rats and alcohol-dependent people. The impaired GABA clearance within the CeA contributes to alcohol addiction, and may be a target for new pharmacotherapies.

Glutamate and GABA exert interacting effects, including not only inverse synaptic signaling but also bioconversion in the brain. Endogenous brain GABA influences the glutamate–glutamine cycling flux. In astrocytes, glutamate and GABA are converted to glutamine through the glutamine synthetase pathway. After being transferred back to neurons, glutamine is converted to glutamate by glutaminase. Meanwhile, glutamate is the precursor to GABA and is back-converted to GABA *via* glutamic acid decarboxylase. Glutamate and GABA are important therapeutic targets in alcohol use disorder. Several glutamate system modulators (acamprosate, gabapentin, and topiramate) and GABA system modulators (benzodiazepines, baclofen, and sodium oxybate) have been recommended as pharmacotherapy for patients with alcohol use disorder (11, 12). Considering the prominent and interacting effects of glutamate and GABA in alcohol use disorder, there is a need to summarize and review the current research status in this domain.

The number of publications in journals is expanding exponentially over time. It is becoming more and more difficult to identify the research frontier and extract the key knowledge nodes of a certain subject or domain. Over the past two decades, bibliometric analysis has become an emerging statistical and visualization tool. It focuses on the impacts of publications, the contributions of individuals, institutes and countries, as well as research frontiers and hotspots in the field (13). Citespace and WOSviewer are two of the most popular bibliometric software packages. CiteSpace is a free Java-based bibliometric software package developed by Dr. Chaomei Chen (14). It provides multiple functions for bibliometric studies including collaboration network analysis, co-citation analysis, and co-occurrence analysis (15). A series of knowledge maps generated by Citespace helps one to explore the research frontiers and evolution of a scientific domain, and reveals the collaboration characteristics of institutions and authors in research fields (16). WOSviewer, created by Leiden University's Center for Science and Technology Studies, is another popular and freely available software package for science mapping (17). It enables network layout and network clustering, and provides integrated visualization of scientific maps. A new open-source tool, *bibliometrix*, developed by Dr. Massimo Aria and Dr. Corrado Cuccurullo (18), is programmed in R and can be integrated with other statistical R-packages. *Bibliometrix* provides a set of tools for quantitative analyses and visualization in bibliometrics and scientometrics.

Considering that studies on the involvement of glutamate and GABA in alcohol use disorder have developed rapidly over the past two decades, it is necessary to summarize the literature characteristics and research direction as a whole, to explore how the research has changed over time, and to predict future trends in this domain. However, there is no bibliometric analysis of the literature in this field. Accordingly, we conducted a bibliometric analysis of publications since 2005 to provide an overview of documents and explore the hot topics and emerging trends in this field.

## Materials and methods

### Data sources and search strategy

Data were retrieved from two large, multidisciplinary citation databases, Scopus and the Web of Science Core Collection (WoSCC). The search phrases associated with the neurotransmitters glutamic acid and GABA included gamma-aminobutyric acid, GABA, glutamate, glutamine, and glutamic acid, and the phrases associated with alcohol use disorder included alcohol use disorder, alcohol abuse, alcohol dependence, alcoholi^*^, and alcohol addiction. The WoSCC includes cited references from 2005. Thus, the search time span was set as the years 2005–2021. Only two types of document, articles and reviews, were included to better represent the research field. The publication language was restricted to English. The same search strategy was applied to both databases. Raw data including full records and cited references were downloaded from Scopus and WOSCC as ris or txt file required for further processing in CiteSpace.

### Data analysis

The data were obtained on April 22nd, 2022 from different databases. Data cleaning was conducted using CiteSpace software in combination with manual searching.

In this study, R–*bibliometrix* was used to perform descriptive analysis of the leading research authors, countries, and journals, identify core journals, and produce a country/region collaboration map. Bradford's law (19, 20) was used as an objective measurement for core journals. In the collaboration map within countries/regions, the darkness of color represents the number of publications from different countries/regions, and the thickness of lines between two countries/regions suggests the intensity of collaborations between individual countries/regions.

CiteSpace 6.1.R1 was employed to detect the collaboration characteristics of institutions and authors, the reference co-citation network and keywords citation bursts. The parameters of CiteSpace were set as follows: time-slicing was performed from January 2005 to December 2021 (1–2 years per slice); the strength of links was calculated based on the Cosine algorithm; the selection was based on a modified g-index with a scale factor k = 25 for collaboration detection, and the top 50 items were selected for keyword analysis and reference co-citation analysis; a pruning with minimum spanning tree was used for concurrence analysis. Nodes and links were used to generate visualization knowledge maps. The size of nodes represents the number of citations. The color of rings indicates citation years: the transition from cool to warm represents early to recent publications. A link between two nodes suggests a co-occurrence or co-citation relationship. Line thickness represents the strength and the color corresponds to the first co-occurrence or co-citation time. In addition to the number of publications and citations, “centrality” was used as a measurement of significance. “Centrality” detects the interactions of a node with other nodes. Nodes with high centrality are considered as an important “bridge” between different groups and represent turning points or pivotal points in this field. Nodes with centrality > 0.1 were surrounded by a purple ring. Co-occurrence analysis of keywords was conducted using WOSviewer 1.6.18. Keywords occurring more than ten times were presented in three visualizations (network, overlay, and density visualization) to identify important terms.

## Results

### General characteristics of publication outputs and sources

**Figure 1:**
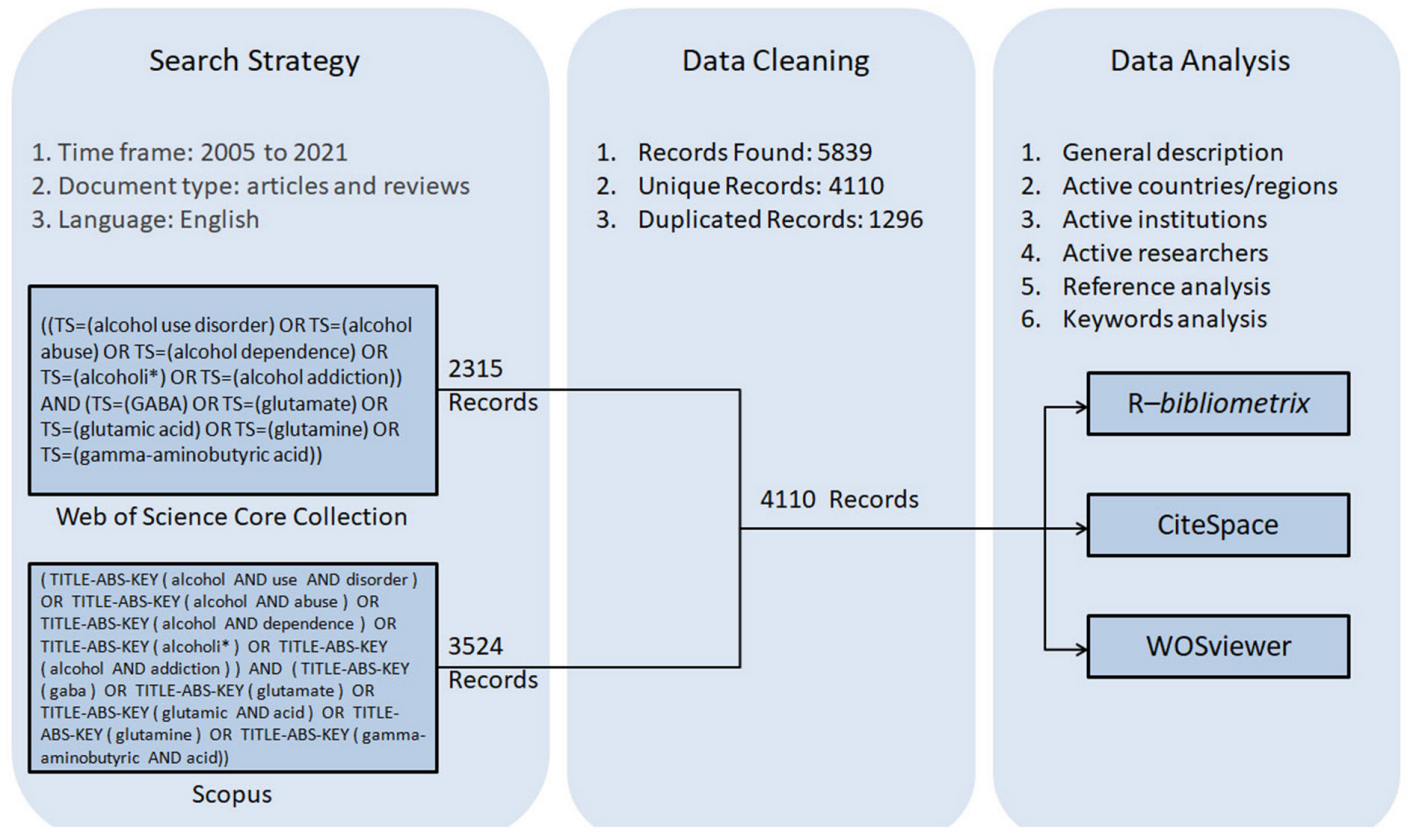
Flowchart for searching, cleaning, and analysis of literature.

**Figure 2:**
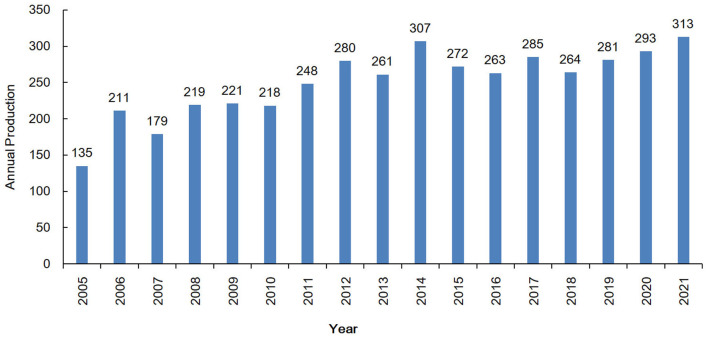
Annual production with regard to the involvement of glutamic acid and GABA in alcohol use disorder.

The total numbers of documents in the Scopus and WoSCC databases were 3,524 and 2,315. Duplicated records were identified and removed. A total of 4,250 unique records from Scopus and WoSCC, including 3,817 primary research articles and 433 reviews, were used for further analysis (Figure 1). The total number of cited references was 222,734. The life span of these documents was 2005–2021. Publication outputs showed a fluctuating growth trend, with an annual growth rate of 5.4% from 2005 to 2021 (Figure 2).

The total number of citations has increased in the past 17 years. Seventeen articles were qualified as “citation classics” in this area, i.e., having 400 or more citations (21). The most frequently cited document was The International Classification of Headache Disorders by Olesen et al. (22), and the most co-cited document was Neurocircuitry of Addiction by Koob et al. (23). These findings indicate a sustained interest in the involvement of glutamic acid and GABA in alcohol use disorder.

The literature was published in 1,248 sources. The journal with the highest number of publications was *Alcoholism: Clinical and Experimental Research* (260 publications, IF_2020_ = 3.455). After Bradford's law was applied, the core distribution comprised 22 journals (Table 1). Most of the top journals were specialized neuroscience or substance abuse journals. The top journals belong to the substance abuse category. These journals are mostly located in the United States (USA) and the United Kingdom (UK). The impact factor of *Biological Psychiatry* (36 publications, IF_2020_ = 13.382) and *Proceedings of the National Academy of Sciences of the United States of America* (27 publications, IF_2020_ = 11.205) exceeds 10.

**Table 1:** Core journals of glutamate and GABA involved in alcohol use disorder.

**Rank**	**Journal**	**Publications**	**Citations^a^**	**Country/region**	${\text{IF}}_{2020}^{\text{b}}$	**Category and JCI quartile**
1	Alcoholism: Clinical and Experimental Research	260	10468	USA	3.455	Substance Abuse, Q2
2	Addiction Biology	125	1999	England	4.28	Biochemistry & Molecular Biology, Q1; Substance Abuse, Q1
3	Alcohol	100	2572	USA	2.405	Pharmacology & Pharmacy, Q2; Substance Abuse, Q3; Toxicology, Q2
4	Neuropharmacology	99	2858	England	5.251	Neurosciences, Q1; Pharmacology & Pharmacy, Q1
5	Neuropsychopharmacology	99	4144	England	7.855	Neurosciences, Q1; Pharmacology & Pharmacy, Q1; Psychiatry, Q1
6	Psychopharmacology	99	5324	Germany	4.53	Neurosciences, Q2; Pharmacology & Pharmacy, Q1; Psychiatry, Q1
7	Alcohol and Alcoholism	62	2025	England	2.826	Substance Abuse, Q3
8	Pharmacology Biochemistry and Behavior	62	2596	England	3.533	Behavioral Sciences, Q2; Neurosciences, Q2; Pharmacology & Pharmacy, Q2
9	Journal of Neuroscience	55	7248	USA	6.167	Neurosciences, Q1
10	Plos One	52	1494	USA	3.24	Multidisciplinary Sciences, Q1
11	Behavioral Brain Research	42	1136	Netherlands	3.332	Behavioral Sciences, Q2; Neurosciences, Q2
12	Frontiers in Psychiatry	39	299	Switzerland	4.157	Psychiatry, Q2
13	Neuroscience	38	2203	England	3.59	Neurosciences, Q2
14	Drug and Alcohol Dependence	37	1704	Switzerland	4.492	Psychiatry, Q1; Substance Abuse, Q1
15	Biological Psychiatry	36	2863	USA	13.382	Neurosciences, Q1; Psychiatry, Q1
16	Brain Research	33	2674	Netherlands	3.252	Neurosciences, Q3
17	Current Pharmaceutical Design	32	203	U Arab Emirates	3.116	Pharmacology & Pharmacy, Q3
18	Neuroscience Letters	31	1103	Netherlands	3.046	Neurosciences, Q3
19	Journal of Psychopharmacology	27	394	England	4.153	Clinical Neurology, Q2; Neurosciences, Q2; Pharmacology & Pharmacy, Q2; Psychiatry, Q2
20	Neuroscience and Biobehavioral Reviews	27	748	England	8.989	Behavioral Sciences, Q1; Neurosciences, Q1
21	Proceedings of the National Academy of Sciences of the United States of America	27	3064	USA	11.205	Multidisciplinary Sciences, Q1
22	Frontiers in Neuroscience	26	284	Switzerland	4.677	Neurosciences, Q2

a *cited by reference*.

b *impact factor in category according to Journal Citation Reports (2020) by Clarivate*.

### Active countries/regions, institutions, and researchers

**Figure 3:**
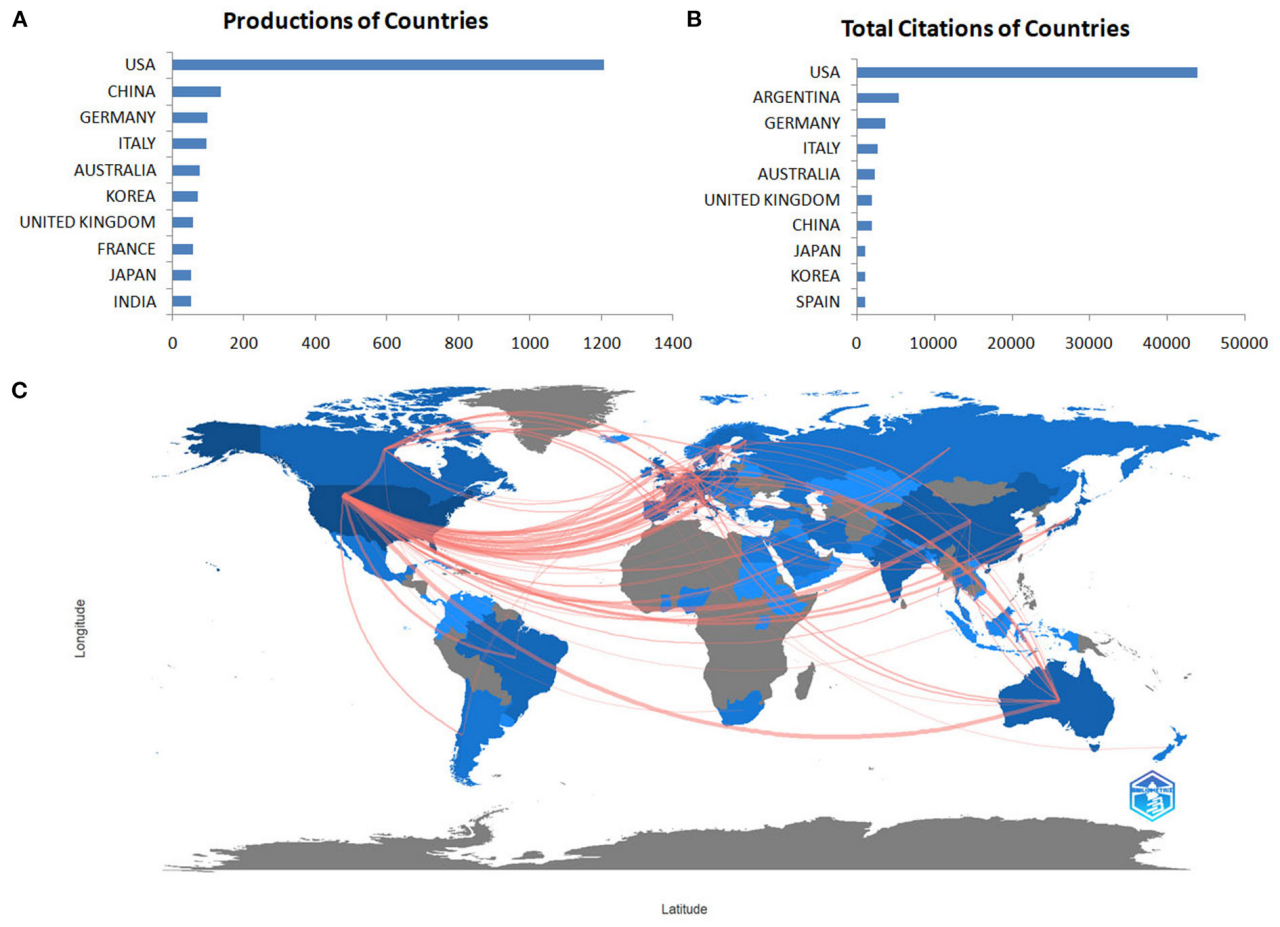
The number of documents **(A)**, citations **(B)** and cooperation networks **(C)** of the active countries/regions.

Eighty-one countries or regions have contributed to publications in the field of research on the involvement of glutamate and GABA in alcohol use disorder. The data extracted from Scopus and WoSCC indicated that the USA was the most productive country (52%), followed by the People's Republic of China (6%), Germany (4%), Italy (4%), and Australia (3%). The centralities of the USA (1.03), Italy (0.15), France (0.13), Canada (0.13), Ukraine (0.12), and the Russian Federation (0.12) are > 0.1, indicating that these countries might deeply cooperate with other countries and act as important intermediaries in this field. Figure 3 displays the numbers of documents and the cooperation networks of the active countries/regions. There were 279 collaborating countries/regions worldwide, of which the top three were USA–Italy, followed by USA–China and USA–Australia.

**Figure 4:**
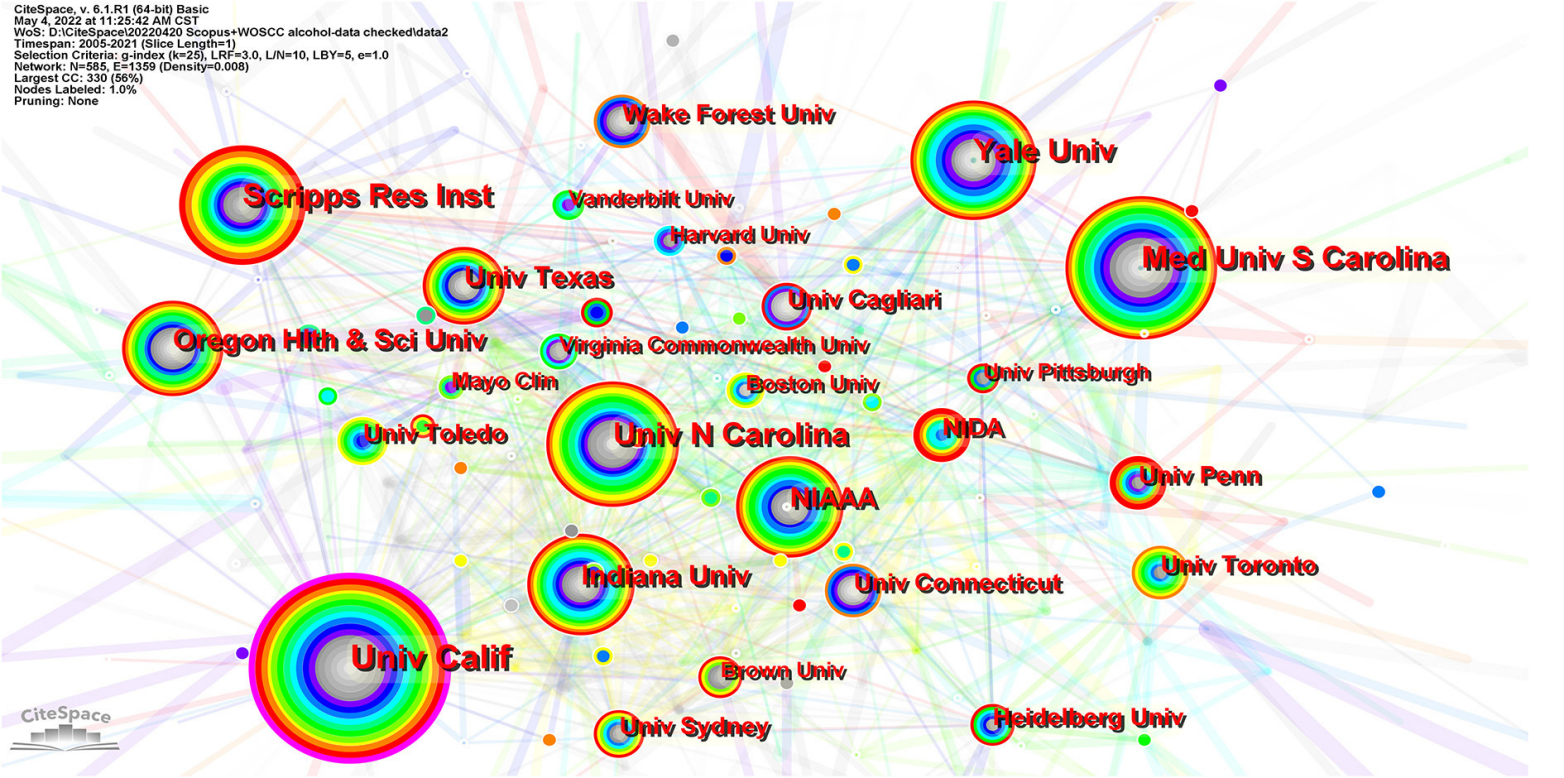
The major productive co-institutions in this field. Each node represents one institute. The size of a node represents the number of citations, and the color indicates citation years, from cool to warm representing early to recent. The links suggest collaborative relationships between institutions. Significant nodes are surrounded by a purple ring.

From 2005 to 2021 3,626 institutions in this field published papers. Table 2 lists the top 10 institutions based on publications and centrality. Figure 4 exhibits the major productive co-institutions in this field. The University of California is the most influential institute in this field, with a total number of 238 publications and centrality of 0.16, followed by the Medical University of South Carolina and Yale University. The top ten productive institutions in this research field are all located in the USA.

**Table 2:** The top ten institutions related to glutamate and GABA in alcohol use disorder.

**Rank**	**Institution**	**Number of publications**	**Centrality**	**Country/region**
1	Univ Calif	238	0.16	USA
2	Med Univ S Carolina	163	0.07	USA
3	Yale Univ	155	0.08	USA
4	Scripps Res Inst	152	0.06	USA
5	Univ N Carolina	238	0.04	USA
6	NIAAA	80	0.04	USA
7	Oregon Hlth & Sci Univ	121	0.03	USA
8	Indiana Univ	170	0.04	USA
9	Univ Texas	117	0.04	USA
10	Univ Connecticut	67	0.03	USA

**Figure 5:**
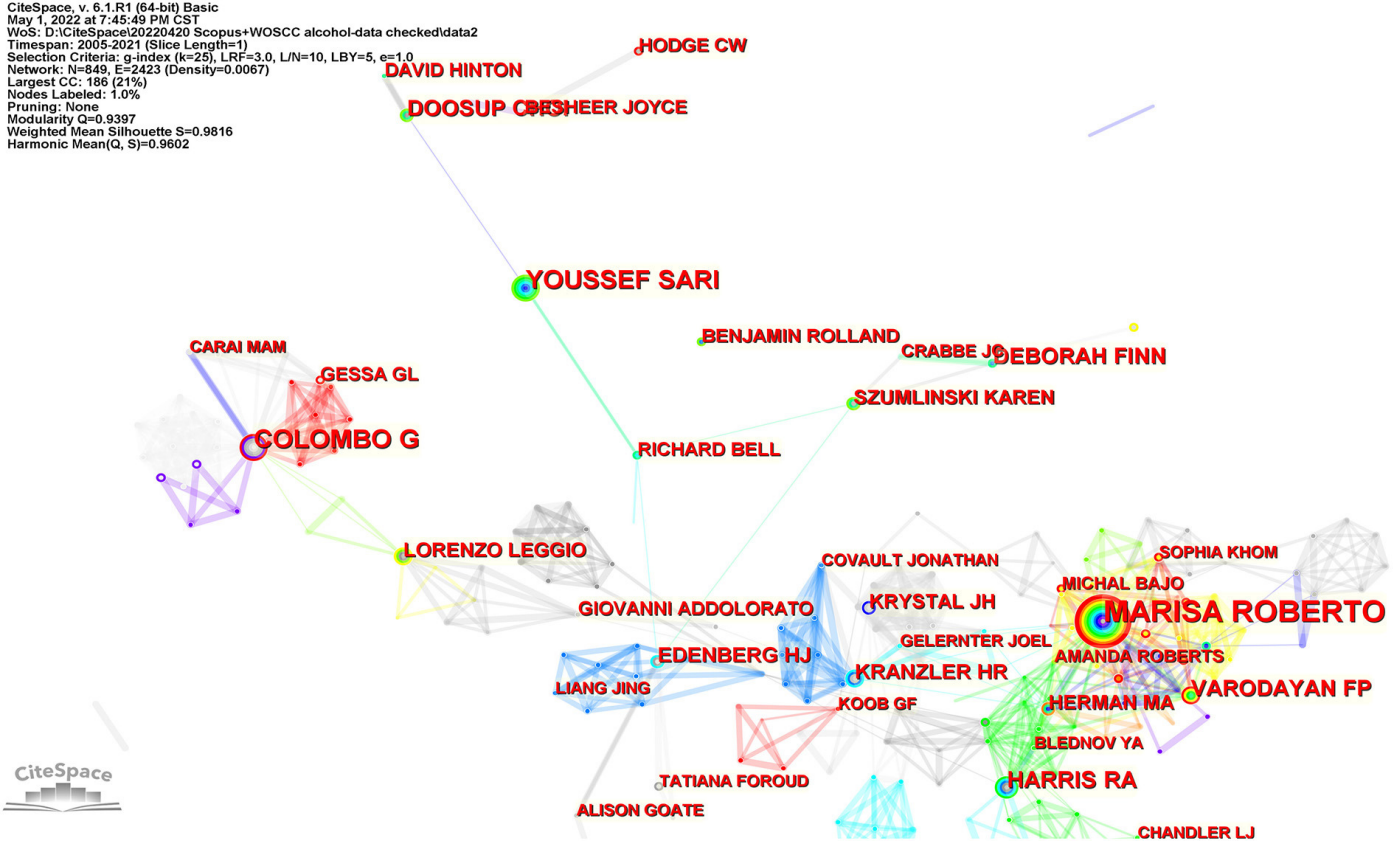
The major productive authors in this field. Each node represents one author. The size of a node represents the number of citations, and the color indicates citation years, from cool to warm representing early to recent. The links suggest collaboration relationships between authors.

A total of 18,145 authors contributed relevant publications. Figure 5 shows the co-authorship network map. Marisa Roberto from the Scripps Research Institute was the most prolific author (57 documents), with the highest centrality of 0.06. The other productive researchers were Giancarlo Colombo (National Research Council, Italy) and Youssef Sari (University of Toledo, USA). Table 3 lists the top 10 productive authors in this research field, their affiliations and centralities.

**Table 3:** The top ten authors related to glutamate and GABA in alcohol use disorder.

**Rank**	**Author**	**Number of publications**	**Centrality**	**Institution**
1	Roberto M	57	0.06	Scripps Res Inst
2	Colombo G	36	0.02	National Research Council
3	Sari Y	32	0.01	University of Toledo
4	Harris R	27	0.01	University of Texas
5	Choi D	21	0.01	Mayo Clinic
6	Varodayan F	21	0.00	Scripps Research Institute
7	Finn D	23	0.01	Oregon Health & Science University
8	Kranzler H	29	0.04	University of Pennsylvania
9	Edenberg H	22	0.01	Indiana University
10	Szumlinski K	27	0.01	University of California

### Analysis of co-cited references

The top co-cited references with the most citation counts and highest centralities are shown in Table 4. According to the ranking of frequency and centrality in the co-cited references, half were review papers, and a few were original research papers. The first ranked citation in terms of frequency was the review published in *Neuropsychopharmacology* titled “Neurocircuitry of addiction” (23). In this review, Koob et al. conceptualized drug addiction as an addiction cycle composed of three stages: “binge/intoxication,” “withdrawal/negative affect,” and “preoccupation/anticipation” (craving). He described discrete circuits involved in these three stages, and that neuroplasticity in these structures contributed to the transition to addiction. The co-cited reference with the highest centrality was an article published in *Neuroscience* by Sari et al. (24). In this article, the authors found that neuroimmunophilin GPI-1046 attenuates ethanol intake in part through the upregulation of GLT1 in the pre-frontal cortex (PFC) and nucleus accumbens core (NAc) of alcohol-preferring rats.

**Table 4:** The top ten co-cited references.

**Rank**	**Local citations**	**Representative author**	**Publication year**	**Journal**	**DOI**	**Title**
1	160	Koob G	2010	Neuropsychopharmacol	10.1038/NPP.2009.110	Neurocircuitry of addiction
2	135	Edenberg H	2004	Am J Hum Genet	10.1086/383283	Variations in GABRA2, encoding the alpha 2 subunit of the GABA(A) receptor, are associated with alcohol dependence and with brain oscillations
3	117	Gass J	2008	Biochem Pharmacol	10.1016/J.BCP.2007.06.039	Glutamatergic substrates of drug addiction and alcoholism
4	115	Lovinger D	1989	Science	10.1126/SCIENCE.2467382	Ethanol inhibits NMDA-activated ion current in hippocampal neurons
5	110	Kumar S	2009	Psychopharmacology	10.1007/S00213-009-1562-Z	The role of GABAA receptors in the acute and chronic effects of ethanol: a decade of progress
6	107	Grobin A	1998	Psychopharmacology	10.1007/S002130050685	The role of GABAA receptors in the acute and chronic effects of ethanol
7	106	Addolorato G	2002	Alcohol Alcoholism	10.1093/ALCALC/37.5.504	Baclofen efficacy in reducing alcohol craving and intake: a preliminary double-blind randomized controlled study
8	106	Kalivas P	2009	Nat Rev Neurosci	10.1038/NRN2515	The glutamate homeostasis hypothesis of addiction
9	102	Roberto M	2004	J Neurosci	10.1523/JNEUROSCI.3004-04.2004	Cellular/molecular increased GABA release in the central amygdala of ethanol-dependent rats
10	99	Roberto M	2003	P Natl Acad Sci USA	10.1073/PNAS.0437926100	Ethanol increases GABAergic transmission at both pre- and postsynaptic sites in rat central amygdala neurons

**Figure 6:**
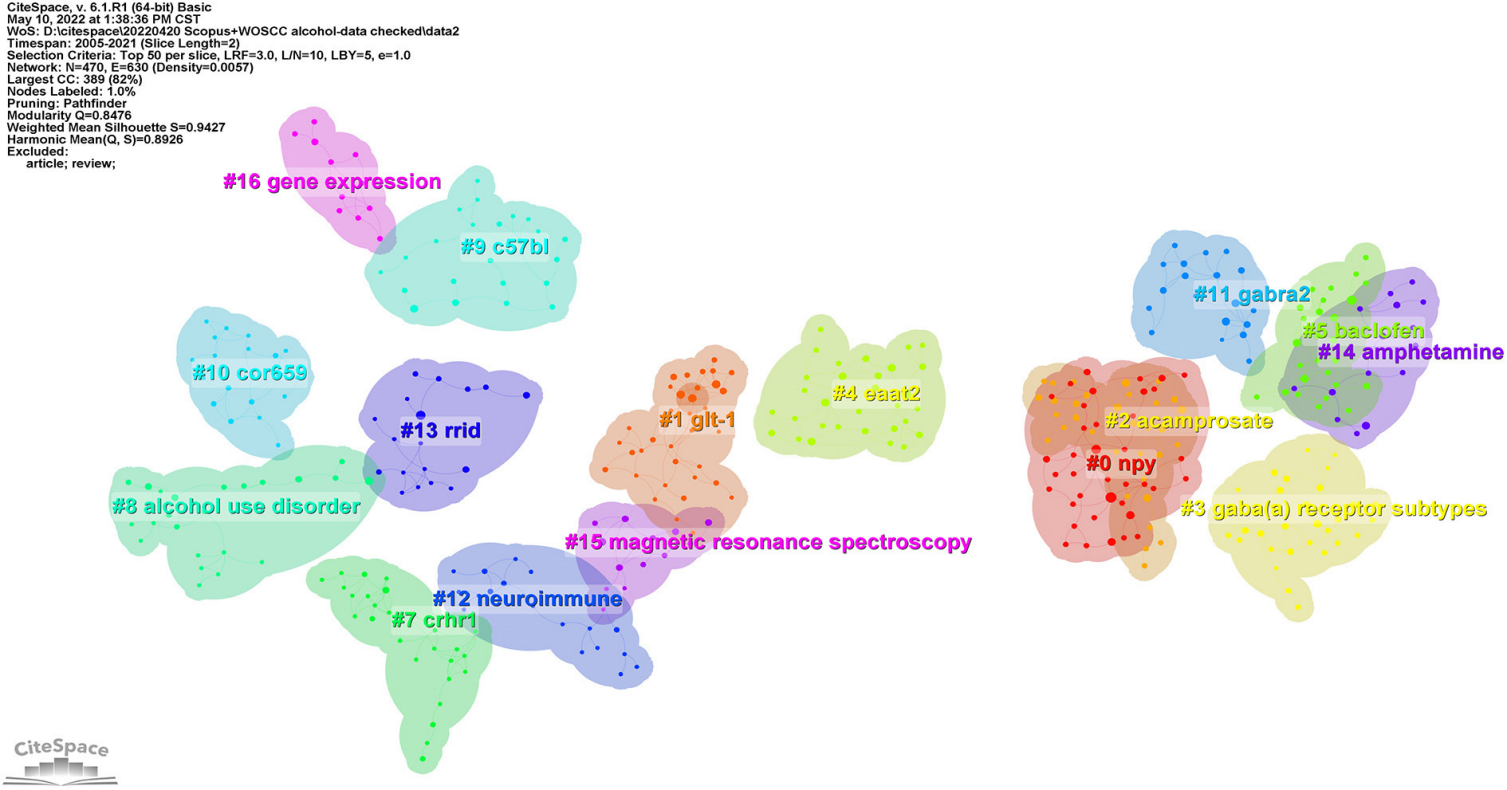
The network map of the cited references.

The network map of the co-cited references is displayed in Figure 6. The reference co-citation network was divided into 17 co-citation clusters. The clusters are labeled by keywords of the cited articles using the LLR (log-likelihood ratio) algorithm as the extraction method. In the study period, the labels of the top ten clusters were: #0 NPY, #1 GLT-1, #2 acamprosate, #3 GABA_A_ receptor subtypes, #4 EAAT2, #5 baclofen, #7 corticotropin releasing hormone receptor 1 (CRHR1), #8 alcohol use disorder, #9 C57BL/6, #10 COR659 (a GABA_B_ positive allosteric modulator). In the research period, a comprehensive analysis referring to multiple studies mainly focused on the following: (1) mechanism, including modulators, transporters, receptor subtypes, and the development of animal models; (2) drug development targeting glutamate and the GABA system for the treatment of alcohol use disorder, including acamprosate, baclofen and COR659.

### Analysis of keywords

**Figure 7:**
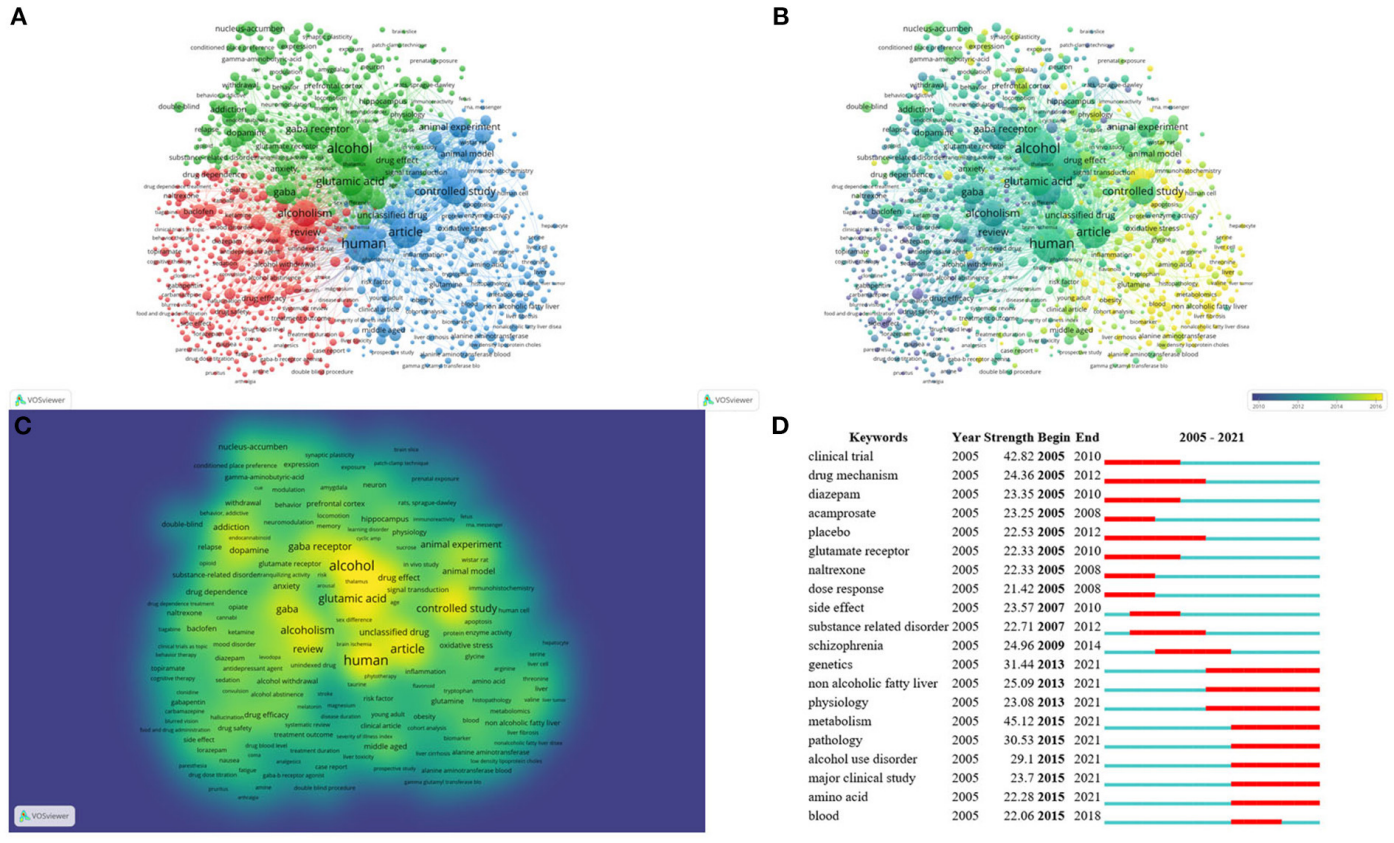
Keyword analysis. Co-occurrence analysis of keywords by WOSviewer, including **(A)** cluster visualization for keywords of studies, **(B)** time trend visualization of keywords (average publication year from blue to yellow indicating from earlier to later), and **(C)** density visualization of keywords (keywords in yellow represent the highest frequency). Also shown are the top 20 keywords with the strongest citation bursts according to Citespace **(D)**, in which the blue part indicates the time interval, and the red part indicates the duration of the citation burst.

A keyword co-occurrence knowledge map was generated by WOSviewer (Figure 7). The font size of the keywords represents their frequencies in records. The highest landmark nodes, such as human, non-human, clinical trial, controlled study, and animal experiment, represented the objects and methods of research. The keywords that occurred more than ten times were organized into three clusters (Figure 7). The three most frequently used keywords were “human,” “alcohol,” and “nonhuman.” In cluster 1, “alcoholism” is the largest node, and includes review, baclofen, drug efficacy, benzodiazepine, naltrexone, placebo, acamprosate, clinical trial, diazepam, dose response, treatment outcome, drug safety, benzodiazepine derivative, gabapentin, and topiramate. Cluster 1 focuses on clinical studies intended to investigate therapeutic medications for alcohol use disorder. In cluster 2, “alcohol” is the largest node. The other main node includes non-human, glutamic acid, GABA, GABA receptor, addiction, brain, dopamine, NAc, cocaine, NMDA receptor, PFC, hippocampus, and ventral tegmental area. Cluster 2 focuses on neurotransmitter transmission in different brain regions and explores relevant mechanisms for alcohol use disorder. In cluster 3, “human” is the largest node and includes controlled study, metabolism, unclassified drug, genetics, protein expression, oxidative stress, middle-aged, gene expression, signal transduction and non-alcoholic fatty liver. Cluster 3 represents the current research hotspot related to risk factor/biomarker identification and drug development for alcohol use disorder and mechanisms related to alcoholic fatty liver.

A map based on the top 20 keywords with the strongest citation bursts was generated by Citespace (Figure 7). The noticeable keywords in the early stage (2005–2012) included clinical trial, drug mechanism, diazepam, acamprosate, placebo, glutamate receptor, naltrexone, and dose response. In recent years (2013–2021), genetics, non-alcoholic fatty liver, physiology, metabolism, pathology, alcohol use disorder, major clinical study, amino acid, and blood were the main research frontiers. The early stage of research mainly focused on drug development for alcohol-related disorders and mechanisms underlying the involvement of glutamic acid and GABA in alcohol use disorder. The research frontiers are shifting to risk factor/biomarker identification, drug development and the pathology of alcohol use disorder, and the genetics, physiology, pathology and amino acid metabolism of related liver disease.

## Discussion

### General trends

In this study, bibliometric analyses and network visualizations were conducted to characterize the knowledge domains of glutamate and GABA related to alcohol use disorder. The contributions of countries, institutions, journals, and authors to this field were analyzed. Research frontiers and hot topics in the coming years were also identified. Since 2005, the annual publication number in the field has increased steadily.

The USA has the largest number of publications and citations, the strongest collaborations worldwide, and the highest centrality among countries/regions. The following countries, China, Germany, Italy, Australia, and Korea, accounted for 20% of all studies included, whilst Argentina, Germany, Italy, Australia and the UK constituted 22% of total citations. China ranked second in the total number of publications, but ranked seventh in the total number of citations and ninth in collaboration with other countries/regions, reflecting China's lack of international cooperation in research. The top ten most productive institutions are located in the USA. Therefore, the USA is currently the world leader in this research domain, and has a significant impact on the direction of research in this field.

### Influential authors and affiliations

Three of the most prolific authors were Marisa Roberto (Scripps Research Institute, USA), Giancarlo Colombo (National Research Council, Italy), and Youssef Sari (University of Toledo, USA). Each of them was in discrete collaborative relationships. Marisa Roberto, Florence P. Varodayan, Amanda Roberts, Sophia Khom, and Michal Bajo, from the Scripps Research Institute, formed a close collaborative relationship, in which Melissa Herman from the University of North Carolina, R. Adron Harris, and Blednov Y.A. from the University of Texas participated. With the largest number of documents and the highest number of citations, Dr. Marisa Roberto is the most important researcher in this collaboration. Marisa Roberto identified the key role of the CeA in alcohol use disorder and that aberrant amygdala GABA transmission was associated with alcohol use disorder (25). Her most cited article was about the key role of CRF-induced amygdala GABA release in alcohol dependence (26). She has recently focused on the impact of interleukin (IL) on CeA transmission (27, 28) and the sex difference (29) associated with alcohol use disorder.

Giancarlo Colombo, Gian Luigi Gessa, and Mauro A. M. Carai, affiliated to the National Research Council of Italy, form a close collaborative relationship. Dr. Giancarlo Colombo is the most important researcher in this collaboration. He mainly focuses on the role of positive allosteric modulators targeting the GABA_B_ receptor in drinking behavior. His most co-cited document was about baclofen-induced reduction of alcohol reinforcement in alcohol-preferring rats (30). Recently, he has focused on preclinical investigations of novel positive allosteric modulators of the GABA_B_ receptor as treatments for alcohol use disorder (31, 32).

Youssef Sari (University of Toledo), Karen Szumlinski (University of California), Deborah Finn (Oregon Health &Science University), Howard J. Edenberg (Indiana University), Richard L. Bell (Indiana University), and Liang Jing (University of Southern California) are in a loose collaborative relationship, in which Youssef Sari is the most important researcher. Dr. Youssef Sariis concerned with the association between brain glutamate homeostasis and substance abuse, and evaluates medications such as antibiotics targeting glutamate transporters to reduce ethanol consumption. His most co-cited document was about the use of ceftriaxone, a beta-lactam antibiotic, as a potential drug to reduce ethanol consumption in alcohol-preferring rats (33). He has recently investigated how antibiotics influence xCT and GLT-1 modulation of glutamate (2, 34).

In addition, Henry R Kranzler (University of Pennsylvania), John Krystal (Yale University), Jonathan M. Covault (UConn John Dempsey Hospital) and Joel Gelernter (Yale University), were in a collaborative relationship. Although the University of California is the most prolific institution, collaboration was not detected among its researchers.

Dr. George F. Koob, director of the National Institute on Alcohol Abuse and Alcoholism (NIAAA) at the National Institutes of Health, is an expert on substance addition not restricted to alcohol abuse. He conceptualized the substance addiction process into three stages (binge/intoxication, withdrawal/negative affect, and preoccupation/anticipation) and three domains of dysfunction (incentive salience/pathologic habits, negative emotional states, and executive function, respectively) *via* changes in the basal ganglia, extended amygdala/habenula, and frontal cortex (23). Koob et al. proposed the term “hyperkatifeia”, defined as a greater intensity of negative emotional/motivational signs and symptoms during withdrawal from drugs of abuse in the withdrawal/negative affect stage of the addiction cycle, providing an additional source of motivation for compulsive drug seeking via negative reinforcement in his latest review (35).

### Outlook

Our co-occurrence network maps, clustered by keywords and co-cited references, indicate that the current hot topics and future directions in the association of glutamate and GABA with alcohol use disorder may be divided into several branches: clinical study and novel drug development; biomarker identification; neuropathology; risk factors. A short synopsis of the main trend topics is shown in Table 5.

**Table 5:** The synopsis of the main trend topics.

(1) Major clinical studies show baclofen as a treatment for alcohol dependence is still premature.
(2) Biomarkers such as gut microbial fingerprint, DNA methylation in GABA receptor genes, and blood metabolites have been identified for alcohol use disorder.
(3) Abnormal glial cell functions are promising neuropathology for alcohol use disorder.
(4) Females are more likely to develop alcohol use disorder.

#### Major clinical studies

Recently, multiple clinical studies have been conducted in different countries to evaluate the efficacy of baclofen in the treatment of alcohol dependence. However, the efficacy of baclofen was questioned. Baclofen did not show superiority over placebo in the maintenance of abstinence, while causing a reduction in alcohol consumption and craving, as observed in the ALPADIR study (36). The efficacy and safety of high doses of baclofen for the treatment of alcohol dependence were also examined in a multicenter, double-blinded, and placebo-controlled trial. The results indicated that large-scale prescription of baclofen for the treatment of alcohol dependence seems premature and should be reconsidered (37). Another multisite, double blind, placebo-controlled, randomized clinical trial indicated that baclofen may be an effective treatment option for patients with alcoholic liver disease (38). However, baclofen is not as good as chlordiazepoxide in the treatment of uncomplicated alcohol withdrawal syndrome (39). Several systematic meta-analyses have been conducted to investigate baclofen as a treatment for alcohol use disorders. Baclofen was associated with higher rates of abstinence than placebo, but showed no superiority in increasing the number of abstinent days, or decreasing heavy drinking, craving, anxiety, or depression (40). The long-term utility of baclofen at both normal and high doses in the treatment of alcohol use disorder was also questioned (41). Therefore, the use of baclofen as a treatment for alcohol dependence is premature and needs to be confirmed in large-scale clinical trials.

#### Biomarkers

Several potential blood biomarkers for alcohol use disorder have been found. Liu et al. (42) conducted an epigenome-wide association study of the methylation of cytosine-phosphate-guanine dinucleotide sites in 13 population-based cohorts and identified an alcohol-related DNA methylation signature. Differential methylation in two GABA receptor genes (GABA_A_δ and GABA_B_ subunit 1) was found. Analysis of DNA methylation may be a promising diagnostic test for heavy drinking. Harada et al. (43) identified nineteen metabolites associated with alcohol intake, and three biomarker candidates (threonine, guanidinosuccinate, and glutamine) for alcohol-induced liver injury by using metabolomics, indicating that the glutamate/glutamine ratio might be a good biomarker for alcohol-induced liver injury. Hyperhomocysteinemia is associated with liver and metabolic diseases. Small heterodimer partner (SHP) inhibits the transcriptional activation of betaine-homocysteine S-methyltransferase and cystathionine γ-lyase by FOXA1. Disruption of SHP in mice alters the timing of expression of genes that regulate homocysteine metabolism and the liver responses to ethanol and homocysteine (44).

Patients with alcohol use disorder present some types of specific gut microbial fingerprint. Alcohol induces gut dysbiosis in human patients and rodent models with alcohol use disorder. Changes in the gut flora were correlated with increased impulsivity, vulnerability, and striatal dopamine 1 receptor expression as well as decreased striatal dopamine receptor 2 expression (45). Moreover, the gut microbiota play a critical role in the progression of alcohol-related liver damage, and interactions between the gut microbiome and liver diseases have been found. Addolorato et al. (46) characterized the gut microbial composition and function in patients with alcohol-associated liver disease (AALD). The alcohol use disorder-associated gut microbiota showed an increased expression of GABA metabolic pathways and energy metabolism. In patients with alcohol use disorder, increased endotoxaemia, systemic inflammatory status, and functional alterations may be involved in the progression of AALD and in the pathogenesis of alcohol use disorder. However, the altered microbial communities vary in different studies, probably owing to differences in alcohol administration regimen and concomitant liver disease (47), so the findings need to be verified.

#### Neuropathology

Research on neuropathology associated with alcohol is increasing recently. Astrocytes are critical constitutions of the brain glutamate–glutamine cycle and play an important role in synaptic glutamate homeostasis. The expression of glial fibrillary acidic protein, a biomarker of astrocyte function, alters following short-term and long-term exposure to alcohol (48). Various glutamate transporters (i.e., GLAST/EAAT1, GLT-1/EAAT2 and xCT) expressed on astrocytes are dynamically regulated by acute and chronic alcohol exposure. Adermark et al. (49) reviewed prominent features displayed by astrocytes and how these properties are influenced by acute and long-term alcohol exposure. Microglia is regarded as “cleaner” of the central nervous system. Alcohol activated the microglia and increased expression of multiple pro-inflammatory cytokines such as tumor necrosis factor α and IL-1β, which may enhance alcohol consumption and contribute to the development of alcoholism. IL-1β mediated ethanol-induced neuroinflammation and interacted with ethanol's effects on CeA GABAergic signaling (50). The innate immune system may also be activated when exposed to excessive ethanol. Toll-like receptor 4 and CD14 signaling are important in the effects of acute ethanol exposure on GABAergic transmission in the CeA (51). In summary, glial cells might be an important target for the development of next-generation treatments for alcoholism.

#### Risk factors

Peltier et al. (52) discussed the critical structures and neurotransmitters underlining sex differences in stress-related alcohol use, the involvement of sex and stress in alcohol-induced neurodegeneration, and the role of ovarian hormones in stress-related drinking in a review. Women are generally more likely to drink to regulate negative affect and stress reactivity. Sex differences in the onset and maintenance of alcohol use begin to develop during adolescence, continue to affect alcohol use into adulthood, and may contribute to chronic and problematic alcohol use.

### Limitations

To the best of our knowledge, this is the first bibliometric analysis to investigate the current status and research trend for glutamate and GABA in alcohol use disorder. However, our analyses have some limitations. First, because the publication language was restricted to English, potential studies in other languages were omitted. Second, the analyses might have been affected by the presupposed criteria of selection. Taking keywords analysis for example, the threshold of keyword occurrence was ten and keywords with occurrence smaller than ten were not included in subsequent analysis. Therefore, latest research trends with related keywords were omitted. Third, the analyses were conducted based on the number of publications and citations as well as centralities to date. For some recently published important studies with low citation frequency, their contributions may have been underestimated.

## Conclusion

Our bibliometric analysis showed that glutamate and GABA continue to be of interest in alcohol use disorder. The USA is a major contributor to knowledge in this field, with the highest number of top institutions and collaborations. Marisa Roberto of the Scripps Research Institute is the most productive author from the largest collaborative relationship. George F. Koob of NIAAA is an expert who has made a significant contribution in substance addiction. The keywords indicate that the focus has evolved from mechanisms and medications targeting glutamate and GABA in alcohol use disorder, to novel drug development, risk factor/biomarker identification in alcohol use disorder, and mechanisms of liver diseases. Our study provides valuable information on potential collaborators and institutions in this field and provides an insight into the developing trends, which may guide new directions for further study.

## Data availability statement

The raw data supporting the conclusions of this article will be made available by the authors, without undue reservation.

## Author contributions

ZW and XN designed the study and retrieved the data. ZW performed the statistical analysis and wrote the first draft. XZ made further modifications. DS and YW supervised the whole process and provided modification advice. All authors contributed to the article and approved the submitted version.

## Funding

This work was supported by the Natural Science Foundation of Guangdong Province (grant number 2021A1515011325), Science and Technology Plan Project of Guangzhou (grant numbers 202102080030 and 202201010736), Scientific Research Project of Traditional Chinese Medicine Bureau of Guangdong Province (grant number 20222177), Science and Technology Plan Project of Guangdong Province (grant number 2019B0303160), and Guangzhou Municipal Key Discipline in Medicine (2021-2023).

## Conflict of interest

The authors declare that the research was conducted in the absence of any commercial or financial relationships that could be construed as a potential conflict of interest.

## Publisher's note

All claims expressed in this article are solely those of the authors and do not necessarily represent those of their affiliated organizations, or those of the publisher, the editors and the reviewers. Any product that may be evaluated in this article, or claim that may be made by its manufacturer, is not guaranteed or endorsed by the publisher.
